# Sequential depletion of human serum for the search of osteoarthritis biomarkers

**DOI:** 10.1186/1477-5956-10-55

**Published:** 2012-09-12

**Authors:** Carolina Fernández-Costa, Valentina Calamia, Patricia Fernández-Puente, José-Luis Capelo-Martínez, Cristina Ruiz-Romero, Francisco J Blanco

**Affiliations:** 1 Rheumatology Division, ProteoRed/ISCIII Proteomics Group, INIBIC – Hospital Universitario de A Coruña, 15006, A Coruña, Spain; 2 REQUIMTE, Departamento de Química, Faculdade de Ciências e Tecnologia, FCT, Universidade Nova de Lisboa, Caparica, Portugal; 3 BIOSCOPE Group, Physical Chemistry Department, Science Faculty, University of Vigo at Ourense Campus, Ourense, Spain; 4 CIBER-BBN Instituto de Salud Carlos III, A Coruña, Spain

## Abstract

**Background:**

The field of biomarker discovery, development and application has been the subject of intense interest and activity, especially with the recent emergence of new technologies, such as proteomics-based approaches. In proteomics, search for biomarkers in biological fluids such as human serum is a challenging issue, mainly due to the high dynamic range of proteins present in these types of samples. Methods for reducing the content of most highly abundant proteins have been developed, including immunodepletion or protein equalization. In this work, we report for the first time the combination of a chemical sequential depletion method based in two protein precipitations with acetonitrile and DTT, with a subsequent two-dimensional difference in-gel electrophoresis (2D-DIGE) analysis for the search of osteoarthritis (OA) biomarkers in human serum. The depletion method proposed is non-expensive, of easy implementation and allows fast sample throughput.

**Results:**

Following this workflow, we have compared sample pools of human serum obtained from 20 OA patients and 20 healthy controls. The DIGE study led to the identification of 16 protein forms (corresponding to 14 different proteins) that were significantly (p < 0.05) altered in OA when compared to controls (8 increased and 7 decreased). Immunoblot analyses confirmed for the first time the increase of an isoform of Haptoglobin beta chain (HPT) in sera from OA patients.

**Conclusions:**

Altogether, these data demonstrate the utility of the proposed chemical sequential depletion for the analysis of sera in protein biomarker discovery approaches, exhibit the usefulness of quantitative 2D gel-based strategies for the characterization of disease-specific patterns of protein modifications, and also provide a list of OA biomarker candidates for validation.

## Background

In clinical proteomics, searching for protein biomarkers is currently done using mass spectrometry-based tools coupled to chromatography or to gel electrophoresis techniques. The samples most commonly used for biomarker discovery are plasma and serum, due to their easy availability. It is generally agreed that each of these samples have more than ten thousand different proteins. From such large amount, it is estimated that circa 20 proteins account for more than 95% of the bulk mass of all proteins [1,2]. These large abundant proteins hinder the searching for putative disease biomarkers, as the signals belonging to such proteins mask those from other less abundant, yet more important proteins in terms of information related to diseases. Therefore, removal of the most abundant proteins helps to overcome the aforementioned problem. However, to date there is no universal method to accomplish this task. Therefore, many different protocols to deplete high abundant proteins can be found in literature comprising, but not limited to: (i) HPLC columns containing antibodies to the most abundant proteins, (ii) spin columns, (iii) the use of chemical reagents, (iv) or even sophisticated kits used to compress the dynamic range of the proteins rather than to deplete them [3-5].

Recently, the use of non-expensive chemical reagents as a way to deplete high abundance proteins has attracted the attention of the proteomics community [6,7]. Furthermore, it has been suggested to link the use of such methods to the resulting proteome after depletion. For instance, serum depleted with DTT was found rich in IgG-type proteins and for this reason it has been proposed as an economic method in studies of diseases that are characterized by modifications in the IgG-type protein content, such as multiple myeloma [8]. After depletion, the resulting proteome is investigated for biomarker discovery using different methodologies, including gel electrophoresis. Two-dimensional difference in gel electrophoresis, 2D-DIGE, has emerged has a powerful tool in proteomics. In 2D-DIGE, complex protein samples are labeled with different fluorescent dyes, mixed together and separated in the same gel. This method solves the problem of gel-to-gel variation [9], and allows for high sample throughput as only one gel is needed to perform the comparisons and spot intensities can be normalized against a unique internal standard.

The present work reports for the first time the combination of a chemical sequential depletion method combined with 2D-DIGE for the search of osteoarthritis biomarkers. Osteoarthritis is a degenerative joint disease characterized by cartilage destruction and bone changes, occasionally accompanied by synovial inflammation [10]. It is the most common rheumatic pathology, and estimates are that the number of people with some degree of OA will double over the next three decades [11]. Despite this high prevalence, existing diagnostic tests are very limited and ultimately rely on the subjective description of pain symptoms, stiffness in the affected joints, and radiography [12]. These limitations have increased the interest in identifying new specific biological markers for cartilage degradation, both to facilitate early diagnosis of joint destruction and to improve the prognosis and evaluation of disease progression. Besides the promising results obtained with some cartilage-specific proteins, still useful biomarkers are needed for clinical practice, which could be effective for the development of early diagnostic tools and/or alternative therapies [13]. The method proposed of sequential depletion for biomarker search in serum samples is non-expensive, of easy implementation and allows fast sample throughput.

## Results and discussion

This work illustrates the application of a chemical sequential depletion method in quantitative proteomics studies for biomarker discovery. The search of novel biomarkers is an area in which proteomics has recently emerged as a powerful approach for the detection of new proteins with diagnostic, prognostic or therapy evaluation utilities. In this field, a number of proteomics experiments have been followed in the last years with the aim of characterizing novel molecules with biomarker power for joint diseases, including osteoarthritis (OA) [14]. In the present study, we report the combination of a chemical sequential depletion method combined with 2D-DIGE for the search of OA biomarkers. This method reduces the dynamic range of serum proteins, thus allowing the identification of a higher number of proteins in the samples.

### Sequential depletion of human serum for proteomics analyses

**Figure 1:**
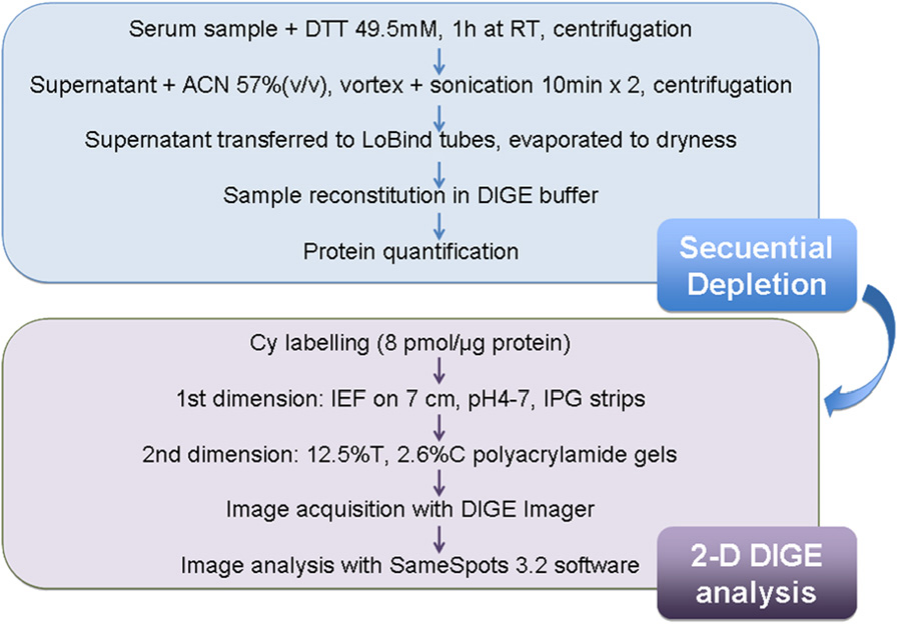
Workflow of the strategy performed for the DIGE-based quantitative differential proteomic analysis on human sera.

We have recently reported the comparison of two different chemical depletion methods, using acetonitrile or DTT, with the protein equalization obtained with the ProteoMiner kit (Bio-Rad) [8] for the mass spectrometry analysis of human serum proteins. Our results showed how ACN depletion was efficient for depleting high molecular weight proteins (over 75 kDa), whereas DTT depletion primarily promotes the precipitation of proteins rich in disulphide bonds (mainly albumin). In the present work, we combine these two chemical depletions in a sequential way, with the aim of simplifying the serum protein profile while removing high abundant (usually uninformative) proteins. The workflow followed for this sequential depletion is showed in Figure 1, and is fully described in the Methods section. Human sera were first depleted with DTT, and the supernatant was further treated with ACN in order to efficiently reduce the dynamic range of proteins in our samples. The protein recovery employing this sequential strategy was 2 ± 0.3%, while the use of either DTT or ACN depletion alone leads to a yield of 32 ± 2% or 4 ± 0.2%, respectively.

**Figure 2:**
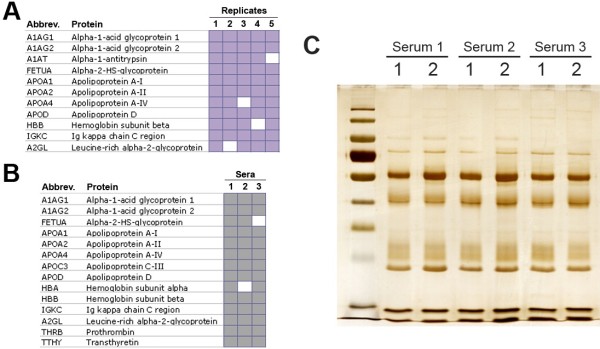
**Reproducibility of the sequential depletion method.** Technical (**A**) and biological (**B**) replicates were analyzed by MALDI-TOF/TOF MS in order to evaluate the reproducibility. Coloured squares show the identification of each protein on the different replicates. (**C**) SDS-PAGE gel (10% acrylamide, silver staining), illustrating the representative protein profiles obtained after the sequential depletion of three independent sera by duplicate, which further demonstrate the reproducibility of the strategy.

To test the reliability of the sequential depletion procedure, we first performed reproducibility tests by analyzing five technical replicates (obtained from five independent depletions of the same serum), and also three biological replicates (obtained from three independent sera). For these analyses, proteins in the depleted samples were fractionated using C18 NuTips (Glygen, Columbia, MD, USA), by eluting with different concentrations of ACN (from 4% to 60%). By these means, ten fractions were obtained from each sample, which were then spotted onto a MALDI plate and analyzed by MS. Figure 2 demonstrates the high technical (A) and biological (B) reproducibility that we found on the sequentially depleted samples, which are also illustrated in the gel shown on Figure 2C.

**Figure 3:**
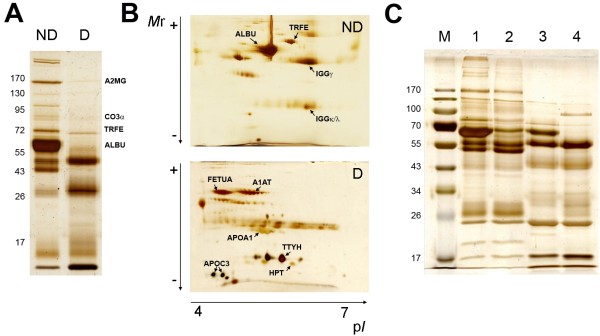
**Differential protein profile of sequentially depleted serum.** One-dimensional (**A**) and two-dimensional (**B**) gel electrophoresis separations, showing the different protein profiles of non-depleted human serum (ND) and serum depleted (**D**) according to the sequential method presented in this work. Some of the most representative proteins that are either depleted or typically present in the depleted maps are depicted. A1AT: alpha-1-antitrypsin; A2MG: alpha-2-macroglobulin; ALBU: serum albumin; APOA1: apolipoprotein A1; APOC3: apolipoprotein C3; CO3α: complement factor α; HPT: haptoglobin; IGGγ: IgG heavy (gamma) chain; IGGκ/λ: IgG light (kappa/lambda) chains; TRFE: transferrin; TTYH: transthyretin. In (**C**), this protein profile is compared to those obtained with ACN and DTT protein depletion methods alone. 1: crude serum, 2: ACN depletion, 3: DTT depletion, 4: sequential depletion.

Proteins in the crude (ND) and depleted (D) samples were separated by one- and two-dimensional gel electrophoresis, in order to evaluate the protein profiles of these two kinds of samples. The different profiles that were obtained after the sequential depletion are illustrated in Figure 3, showing the decrease of high molecular weight proteins that is characteristic of ACN-depleted sera, and also the enrichment of some proteins that were masked by albumin in the crude samples. As shown in the Figure, with this method we were able to deplete in an efficient way serum albumin (ALBU) and other high abundant proteins such as alpha-2-macroglobulin (A2MG), transferrin (TRFE), Complement 3α (C3α), or the heavy chain of IgG (IGGγ).

We randomly picked 54 spots from these 2D gels and identified the protein forms present in each of them, which correspond to 16 different proteins (Additional file 1: Figure S1 and Additional file 2: Table S1). Although many of them belong to the most abundant plasma proteins, as expected for a gel-based technique (which has an important bias towards abundant proteins), we were also able to identify two proteins that are reported to be present at less than 1 μmol/L in plasma, such as serum amyloid A (SAA1) or angiotensinogen (ANGT) [15]. These results support the usefulness of the strategy for the study of medium-abundant serum proteins.

### 2D-DIGE quantitative proteomics analysis on the sequentially depleted serum samples

The sequential depletion method was then applied to study the osteoarthritis-dependent modulation of proteins in serum. Those proteins that are altered in samples from diseased patients might be useful as OA biomarkers for diagnostic or prognostic purposes, and also for the evaluation of alternative therapies.

With this aim, serum samples from 40 patients (20 suffering severe OA and 20 healthy controls) were grouped into four pools of 10 samples each, to reduce the individual and biological variability that has been reported in plasma proteins [16]. Although this involves the loss of each sample individual data, this pooling strategy has demonstrated its usefulness for biomarker discovery in shotgun proteomics approaches [17]. Table 1 summarizes the characteristics of each pool. To perform the differential quantitative analysis, we followed the DIGE strategy [18], which is the best quantitative gel-based approach up to date. It permits direct quantitative evaluation of changes, reduces inter-gel variation and false positives [9,19] and therefore results in highly confident data with biological significance. Moreover, one of the advantages of 2D-DIGE versus non-gel based quantitative proteomics techniques is that it can directly detect not only changes of protein quantity, but also posttranslational modifications of the proteins [20], which are known to be remarkably abundant in serum samples.

**Table 1:** Characteristics of the osteoarthritis (OA) patients and controls included in this study

	**Control**	**OA**
Number of patients	20	20
Age^a)^	67.75 ± 9.08	75.85 ± 5.00
Protein^b)^	57.68 ± 5.06	56.38 ± 4.92
	**Pool 1**	**Pool 2**
Number of patients	10	10
Age	66.20 ± 8.83	76.10 ± 4.79
Protein	57.69 ± 5.15	54.69 ± 4.50
	**Pool 3**	**Pool 4**
Number of patients	10	10
Age	69.30 ± 9.52	75.60 ± 5.44
Protein	57.66 ± 5.25	58.07 ± 3.83

^a)^ Age shown as years with mean ± SD.

^b)^ Protein shown in μg/μl with mean ± SD.

**Figure 4:**
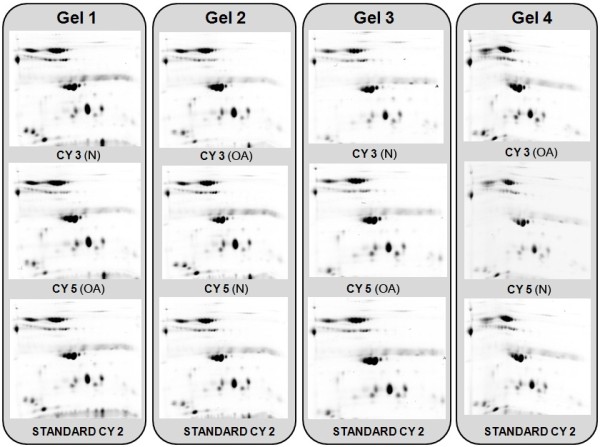
**Images from the DIGE gels obtained in this work.** The two-dimensional gel images corresponding to the Cy2-, Cy3- and Cy5-labeled samples are shown.

The four serum pools were labelled with Cy dyes as described in Table 2. A technical replicate was performed with each sample by swapping the dye (that is, the same sample was labelled once with Cy3 and also with Cy5), in order to avoid labelling artefacts. Sample pairs were combined with the Cy2-labeled internal standard, and proteins were resolved by four-plex 2D gel electrophoresis. The 12 individual images obtained in this experiment (three from each gel, corresponding to Cy2-, Cy3- and Cy5-labeled samples) were analyzed with the SameSpots software, which allowed the normalization of spot intensities and the calculation of abundance ratios and statistical power of the results. All analyzed images are illustrated in Figure 4. An average of 210 protein spots were detected and matched on the gel images. We considered changes within 95% confidence interval (*p* < 0.05) and standardized average spot volume ratios exceeding 1.3.

**Table 2:** DIGE experimental design

**Gel 1**	Pool 1/Cy3	Pool 2/Cy5
**Gel 2**	Pool 1/Cy5	Pool 2/Cy3
**Gel 3**	Pool 3/Cy3	Pool 4/Cy5
**Gel 4**	Pool 3/Cy5	Pool 4/Cy3

### Novel putative serum osteoarthritis biomarkers identified in this work

**Figure 5:**
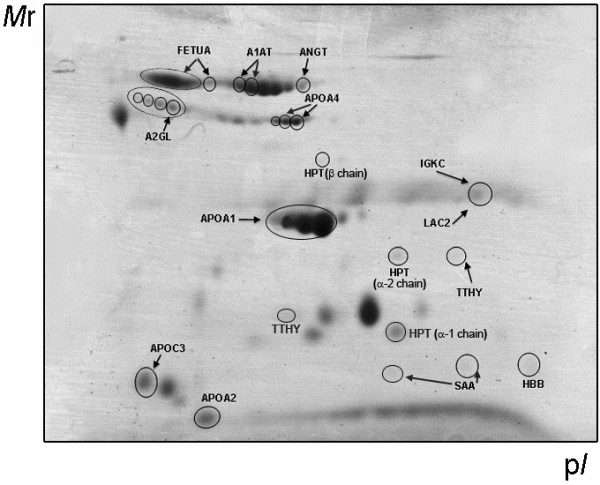
**Two-dimensional gel showing the putative OA protein biomarkers identified in this work.** Protein abbreviations, according to Table 3.

The analysis resulted in 46 spots significantly and reproducibly altered between OA and control samples (29 increased and 17 decreased), which are depicted on the 2D-DIGE gel image shown in Figure 5. For the identification of the altered proteins, spots were excised from the gels, digested in-gel and analysed by MALDI-MS/MS. Mascot database search using the MS/MS spectra allowed the identification of the proteins present in all the spots. Altogether, these 46 spots correspond to 15 different protein forms that were identified as altered in OA. These proteins are listed in Table 3 with complete information comprising experimental and theoretical MW and pI values, accession number and identification parameters. A database search was carried out to assign them into different functional groups, which are also detailed in Table 3.

**Table 3:** Proteins identified in this work as altered in Osteoarthritis vs Control sera

**Protein ID**^**a)**^	**Acc.No.**^**a)**^	**Name**	**Function**	**Ratio OA:N**^**b)**^	**Anova (p)**	**Predicted**^**c)**^		**Experimental**^**d)**^		**Score**^**e)**^	**Sequence tags**^**f)**^
						**MW (Da)**	**pI**	**MW (Da)**	**pI**		
A1AT	P01009	Alpha-1-antitrypsin	Inhibitor of serine proteases	1.4	0.008	46878	5.37	40047	5.33	151	K. TDTSHHDQDHPTFNK.I
											K. ITPNLAEFAFSLYR.Q
											K. QINDYVEKGTQGK.I
											K. GKWERPFEVK.D
											K. DTEEEDFHVDQVTTVK.V
											R. LGMFNIQHCK.K
											K. LQHLENELTHDIITK.F
											K. FLENEDRR.S
											K. VFSNGADLSGVTEEAPLK.L
											K. FNKPFVFLMIEQNTK.S
FETUA	P02765	Alpha-2-HS-glycoprotein	Negative regulation of bone mineralization	2	0.004	40098	5.43	40098	5.43	102	K. CNLLAEKQYGFCK.A
											R. HTFMGVVSLGSPSGEVSHPR.K
ANGT	P01019	Angiotensinogen	Essential component of the renin-angiotensin system (RAS)	1.3	0.001	53406	5.87	40056	5.48	28	K. ANAGKPKDPTFIPAPIQAK.T
APOA1	P02647	Apolipoprotein A-I	Cholesterol metabolism	2.2	0.013	30759	5.56	31221	5.43	271	K. DSGRDYVSQFEGSALGK.Q
											K. LLDNWDSVTSTFSK.L
											K. LLDNWDSVTSTFSKLR.E
											K. VQPYLDDFQKK.W
											K. KWQEEMELYR.Q
											K. WQEEMELYR.Q
											K. VEPLRAELQEGAR.Q
											K. LSPLGEEMRDR.A
											R. THLAPYSDELR.Q
											R. THLAPYSDELRQR.L
											K. ATEHLSTLSEKAKPALEDLR.Q
											K. AKPALEDLR.Q
APOA2	P02652	Apolipoprotein A-II	Lipid transport	0.7	0.003	11282	6.26	11064	5.23	82	K. VKSPELQAEAK.S
											K. SPELQAEAK.S
											K. SKEQLTPLIK.K
APOA4	P06727	Apolipoprotein A-IV	Lipid transport	0.6	0.005	45371	5.28	37738	5.47	37	K. LVPFATELHER.L
											R. LEPYADQLR.T
											R. TQVNTQAEQLRR.Q
											R. QLTPYAQR.M
											K. IDQNVEELKGR.L
											R. ISASAEELRQR.L
											R. LAPLAEDVR.G
											R. LAPLAEDVRGNLR.G
											K. SLAELGGHLDQQVEEFR.R
											K. SLAELGGHLDQQVEEFRR.R
											R. RVEPYGENFNK.A
											K. ALVQQMEQLR.Q
APOC3	P02656	Apolipoprotein C-III	Lipid degradation and transport	0.8	0.014	10846	5.23	13922	5.16	149	K. TAKDALSSVQESQVAQQAR.G
											K. DALSSVQESQVAQQAR.G
HPT	P00738	Haptoglobin (β chain)	Cellular iron ion homeostasis	2	0.007	45861	6.13	35377	5.74	90	K. GSFPWQAK.M
											R. NANFKFTDHLK.Y
											K. YVMLPVADQDQCIR.H
											K. SPVGVQPILNEHTFCAGMSK.Y
HPT	P00738	Haptoglobin (α 1 chain)	Cellular iron ion homeostasis	1.4	0.006	45861	6.13	19281	5.98	68	R. YQCKNYYK.L
											K. LRTEGDGVYTLNNEK.Q
											K. AVGDKLPECEAVCGKPK.N
HPT	P00738	Haptoglobin (α 2 chain)	Cellular iron ion homeostasis	0.5	0.0002	45861	6.13	28110	5.97	135	R. TEGDGVYTLNDKK.Q
											K. LRTEGDGVYTLNNEK.Q
											K. AVGDKLPECEAVCGKPK.N
											K. AVGDKLPECEAVCGKPK.N
HBB	P68871	Hemoglobin subunit beta	Involved in oxygen transport from the lung to peripheral tissues	1.6	0.014	16102	6.75	16102	6.75	43	R. LLVVYPWTQR.F
IGKC	P01834	Ig kappa chain C region	Immune response	0.6	0.003	11773	5.58	33273	6.42	107	-. TVAAPSVFIFPPSDEQLK.S
											K. SGTASVVCLLNNFYPR.E
LAC2	P0CG05	Ig lambda-2 chain C regions	Complement activation, classical pathway	0.6	0.003	11458	6.92	33273	6.42	72	K. AAPSVTLFPPSSEELQANK.A
											K. QSNNKYAASSYLSLTPEQWK.S
											R. SYSCQVTHEGSTVEK.T
											R. SYSCQVTHEGSTVEKTVAPTECS.-
A2GL	P02750	Leucine-rich alpha-2-glycoprotein	Protein binding (secreted)	1.5	0.005	38382	6.45	38507	5.47	88	R. YLFLNGNKLAR.V
											R. VAAGAFQGLR.Q
											R. CAGPEAVKGQTLLAVAK.S
SAA	P02735	Serum amyloid A protein	Acute-phase response	0.8	0.014	13581	6.28	15299	6.53	52	R.EANYIGSDKYFHAR.G
											R.FFGHGAEDSLADQAANEWGR.S
TTHY	P02766	Transthyretin	Thyroid hormone-binding protein	2.2	0.022	15991	5.52	28287	6.3	136	R.KAADDTWEPFASGK.T
											K.AADDTWEPFASGK.T

^a)^ Protein ID and accession number according to SwissProt database.

^b)^ Average volume ratio OA:N, quantified by SameSpots software.

^c)^ Predicted molecular weight (MW) and Isoelectric point (pI) according to protein sequence and SwissProt database.

^d)^ Experimental molecular weight (MW) and Isoelectric point (pI) calculated by analysis of the gel images with SameSpots software.

^e)^ MS protein score obtained from MALDI-TOF/TOF spectra using Mascot search engine.

^f)^ Sequence tags identified by MALDI-TOF/TOF MS/MS.

Interestingly, this altered panel differs substantially from that obtained in a previous work from our group based on serum immunodepletion and subsequent LC-MS/MS analysis [21]. In that work, several relatively high abundant proteins (such as complement components) were found increased in the sera from OA patients. On the contrary, most complement proteins are removed by the different depletion approach in the present study, while several apolipoproteins are identified. This demonstrates the complementarity of different depletion steps. Furthermore, although the gel-based strategy performed in this study did not allowed the identification of less abundant proteins, it demonstrates its usefulness of for characterizing disease-specific patterns of protein modifications, which are impossible to obtain from peptide/MS-based strategies. This includes not only the identification of a specific isoform of HPT beta chain as increased in OA, but also different forms of serum amyloid A, transthyretin or some apolipoproteins.

Nevertheless, both proteomic approaches led to the identification of several ubiquitous proteins related with lipid transport, immune response or protein binding. Although the molecules that had been employed typically as OA biomarkers are proteins directly or indirectly involved in cartilage degradation, or proteins synthesized in an attempt at cartilage repair (including different type II collagen fragments or other cartilage extracellular matrix components) [22], increasing evidences suggest that the most promising strategy in OA would be the combination of different biomarkers into panels. These might include proteins highly specific to the pathology, but also ever-present proteins that can be altered during the progression of several unspecific processes such as inflammation, immune response, cellular death/proliferation, etc. The possible biomarker value of these proteins is exemplified by a recent work unravelling the key role of complement dysregulation in OA pathogenesis [23]. Furthermore, the identification in the present study of several proteins related with the lipid metabolism might be relevant for the definition of a new phenotype termed ‘metabolic OA’, which is recently acquiring more attention in the research community [24].

### Osteoarthritis patients display an altered haptoglobin protein profile in serum

**Figure 6:**
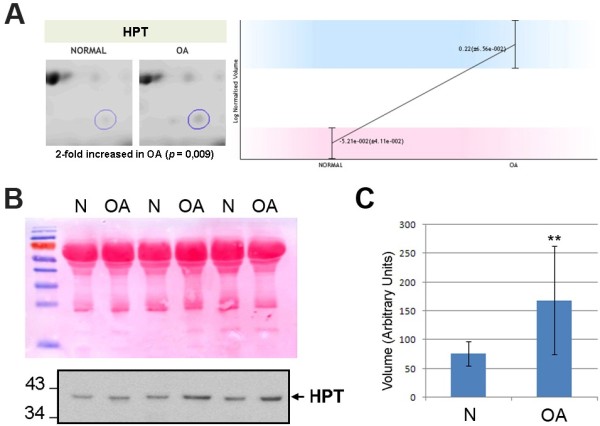
**Verification of the OA-dependent alteration of HPT by Western blot analyses of independent samples.** (**A**) Representative image obtained from the DIGE gels, together with the quantitative data acquired thereof for Haptoglobin beta chain (HPT, which was increased in OA samples). (**B**) Proteins in crude serum samples from OA patients (n = 15) and controls (N, n = 15) were resolved by SDS-PAGE and probed with antibodies against HPT. Band intensities were normalized against the intensity of Ponceau staining (upper figure). (**C**) Semi-quantitative densitometric analysis of the blot signals confirmed the increase of HPT beta chain in OA sera.

Interestingly, we detected three spots corresponding to the same protein accession number that exhibited different alterations in OA sera when compared to control (two increased and the other decreased, with 1.5 to 2-fold variations). They were all identified as haptoglobin precursor (HPT, P00738) in the Mascot search, and the spot that was detected as increased in OA with a MW of around 35 KDa is illustrated in Figure 6A. HPT precursor is an N-linked glycoprotein [25] known to be cleaved into two chains (alpha and beta, see Additional file 3: Figure S2), which combine with free plasma hemoglobin and contribute to the maintenance of cellular iron homeostasis. Previous proteomic studies on synovial fluid, also performed using 2D-gels, reported remarkable interindividual differences in the haptoglobin patterns of the studied patients [26,27]. In the present study, however, we did not analyze individual samples, but pools made from 10 patients each. This strategy has proved its usefulness in our previous iTRAQ-based approach [21], and in the present work it has allowed the detection of changes in HPT chains with a high statistic significance. Nevertheless, we pursued also to verify the alteration of HPT protein pattern in OA sera by an orthogonal strategy, performing Western blot analyses on 30 new individual samples from OA and control donors (n = 15 from each condition). As shown in Figure 6B, a remarkable increase of the HPT beta chain (detected with a molecular weight around 35–40 kDa) can be detected in OA sera. We relatively quantified this increase as 2.22-fold by densitometric analysis of the blots, p = 0.017 (Figure 6C). Although the relationship of HPT polymorphisms with diseases such as cancer or rheumatoid arthritis has been largely studied due to the antioxidant [28], anti-inflammatory [29] and immunomodulatory properties [30] of this protein, this is the first time an alteration of HPT protein pattern is reported in OA.

## Conclusions

In summary, we show in this work the application of a novel sequential depletion procedure for the search of osteoarthritis biomarkers in serum. This strategy has been coupled to a DIGE-based quantitative proteomic analysis, in order to find protein isoforms or fragments that are specific of the disease. By these means, we were able to identify 16 protein forms altered in the disease. Among these, we verified for the first time the OA-dependent increase of an haptoglobin chain. These data demonstrate the usefulness of the approach for protein biomarker discovery, and provide a list of potential OA biomarker candidates that might be subject of further validation studies.

## Methods

### Serum samples

The sera used for this study were obtained from OA patients and controls with no history of joint disease, which were characterized radiographically. Inclusion criteria are fully described in a previous publication from our group [31]. The patients meeting these criteria were diagnosed with OA according to the American College of Rheumatology (ACR) criteria, and knee and hip radiographs from the participants were classified from grade I to grade IV according to the Kellgren and Lawrence (K/L) scoring system [32]. The patients were of both genders and ages ranged from 58 to 90 years. A population of 20 samples from K/L grade IV and 20 non-symptomatic controls was utilized for this study. Prior to proteomic analysis, the serum samples were grouped into four pools of 10 samples each to reduce individual and biological variability. Table 1 summarizes the characteristics of each pool.

### Sequential depletion of high abundant proteins in serum

The sera were subjected to a sequential depletion protocol involving two precipitation steps (Figure 1): protein depletion was first performed with DTT according to the protocol described by Warder et al. [7] with minor modifications, and then further depleted with ACN according to Kay et al. [6]. With this aim, 2.2 μl of 500 mM DTT were added to 20 μl of pooled serum and incubated for 1 h at room temperature until a viscous precipitate persisted. This precipitate was pelleted by centrifugation 2 × 20 min at 14000 × *g*. Subsequently, the supernatant was transferred to a clean LoBind tube and diluted with water prior to the addition of 57% ACN [8]. Samples were briefly vortexed and sonicated twice for 10 min. Then, ACN-precipitated proteins were pelleted during 10 min at 14000 × *g*. Finally, the supernatant was transferred to clean LoBind tubes and evaporated to dryness in a Savant SPD121P SpeedVac (Thermo, Waltham, USA) for further analysis.

### Protein quantification

Precipitated proteins were solubilized in an isolectric focusing-compatible lysis buffer containing 8 M urea, 2 M thiourea, 30 mM Tris and 4% CHAPS. The proteins present in the pools were quantified (n = 5) using the Bradford method (modified with HCl) [33]. A BSA standard curve (0 to 4 mg/ml) and the samples were analyzed in triplicate by reading at 570 nm in a microplate reader (Multiskan® Plus, Labsystems, Quesada, Argentina).

### Protein labeling and two-dimensional electrophoresis

The proteomics comparison between osteoarthritic and control sera was performed across four DIGE gels using 20 μg of total protein fraction per CyDye™ gel and two biological replicates for each condition. Proteins in each sample were fluorescently tagged with a set of matched fluorescent dyes according to the manufacturer’s protocol for minimal labeling. To ensure that there were no dye-specific labeling artifacts, Cy3 and Cy5 labels were swapped between two technical replicates of the same sample, whereas the pooled standard sample was labeled with Cy2. The standard pool was prepared by pooling 20 μg of proteins from each sample prior to labeling. In every case, 160 pmol of dye was used for 20 μg of proteins (8 pmol/μg). Labeling was performed for 30 min on ice in darkness, and the reaction was quenched with 1 μl of 10 mM L-lysine for 10 min under the same conditions.

The four pairs of Cy3- and Cy5-labeled samples (each containing 20 μg of protein) were combined and mixed with a 20-μg aliquot of the Cy2-labeled standard pool. The mixtures, containing 60 μg of protein, were diluted to 125 μl with rehydration buffer (7 M urea, 2 M thiourea, 4% CHAPS, 0.002% bromphenol blue, 2% ampholytes (pH 3–11), and 97 mM Destreak reagent). Samples were loaded onto IPG strips (7 cm, pH 4–7 non-linear) by passive overnight rehydration. Isoelectric focusing was carried out on a IPGphor II IEF system (GE Healthcare) using the following conditions: 333 Vhr at 500 V, 40 min at 1000 V and finally 9166 Vhr at 5000 V. Prior to the second dimension run, the strips were equilibrated first for 15 min in equilibration buffer (100 mM Tris–HCl (pH 8.0), 6 M urea, 30% glycerol, and 2% SDS) with 2% DTT and then for another 15 min in the same buffer supplemented with 2.5% iodoacetamide and 0.002% bromophenol blue. The equilibrated strips were transferred onto 12.5% homogenous polyacrylamide gels (2.6% C) casted in low fluorescence glass plates. Electrophoresis was run at 150 V at 20°C.

### Image acquisition and DIGE data analysis

The differentially labeled co-resolved proteins within each gel were imaged at 100 dots/inch resolution using a DIGE Imager scanner (GE Healthcare). Cy2-, Cy3-, and Cy5-labeled images of each gel were acquired at excitation/emission values of 488/520, 523/580, and 633/670 nm, respectively. Gels were scanned directly between the glass plates, and the 16-bit image file format images were exported for data analysis. After imaging for CyDyes, the gels were removed from the plates and stained with colloidal Coomassie following standard procedures.

Semi-automated image analysis was performed with Progenesis SameSpots V3.2 software (Nonlinear Dynamics). Multiplexed analysis was selected for DIGE experiments and a representative gel image was chosen as reference. Spots were detected and their normalized volumes were ranked on the basis of ANOVA p-values and fold changes.

### Ultrasonic in-gel enzymatic digestion

The gel spots of interest were manually excised and transferred to microcentrifuge tubes. Samples selected for analysis were subjected to ultrasonic in-gel enzymatic digestion, according to the ultrafast proteolytic digestion protocol previously described [34,35]. Briefly, protein bands were washed with water and acetonitrile, reduced with DTT and alkylated with IAA in an ultrasonic bath (Sonorex RK 31 H, Bandelin, Berlin, Germany) operating at 35 kHz (100% amplitude). Then, the gel was washed again, dried and trypsin (375 ng in 25 μl) was added to the dried protein bands. The in-gel protein digestion was performed in a sonicator model SONOPULS HD 2200 (Bandelin, Berlin, Germany) operating at 50% amplitude for 2 min. Finally, supernatants were collected and further peptide extraction was performed on the remaining gel piece with acetonitrile 50% V/V, trifluoroacetic acid 0.1%V/V during 2 min at 50% amplitude in the sonicator. Supernatants were pooled, evaporated to dryness and reconstituted in 5 μl of formic acid 0.3% V/V.

### Mass spectrometry (MS) analysis

The samples were analyzed using the Matrix-assisted laser desorption/ionization (MALDI)-Time of Flight (TOF)/TOF mass spectrometer 4800 Proteomics Analyzer (ABSCIEX, MA, USA) and 4000 Series Explorer™ Software (ABSCIEX). Data Explorer version 4.2 (ABSCIEX) was used for spectra analyses and generating peak-picking lists. All mass spectra were internally calibrated using autoproteolytic trypsin fragments and externally calibrated using a standard peptide mixture (Sigma-Aldrich). TOF/TOF fragmentation spectra were acquired by selecting the 20 most abundant ions of each MALDI-TOF peptide mass map (excluding trypsin autolytic peptides and other known background ions). An average of 2000 laser shots were employed per fragmentation spectrum.

### Database search

The amino acid sequence tags obtained from each peptide fragmentation in MS/MS analyses were used to search for protein candidates using Mascot version 2.2 from Matrix Science (http://www.matrixscience.com). Peak intensity was used to select up to 50 peaks per precursor for MS/MS identification. Tryptic autolytic fragments, keratin and matrix-derived peaks were removed from the dataset used for the database search. The searches for peptide mass fingerprints and tandem MS spectra were performed in the SwissProt knowledgebase (2011_05 release version, June 2011), by searching in the UniProtKB/Swiss-Prot (http://www.expasy.ch/sprot) database, containing 528048 sequences and 186939477 residues, with taxonomy restriction (*Homo sapiens*, 20239 sequences). Fixed and variable modifications were considered (Cys as *S*-carbamidomethyl derivate and Met as oxidized methionine, respectively), allowing one trypsin missed cleavage site and a mass tolerance of 50 ppm. MS identifications were accepted as positive when at least five peptides matched and at least 20% of the peptide coverage of the theoretical sequences matched within a mass accuracy of 50 or 25 ppm with internal calibration. For MS/MS identifications, a precursor tolerance of 50 ppm and MS/MS fragment tolerance of 0.3 Da were used. Probability scores were significant at p < 0.01 for all matches.

### Western blot analysis

One-dimensional Western blot tests were performed according to standard procedures. Briefly, 40 μg of serum proteins were loaded and resolved on standard 15% polyacrylamide SDS-PAGE gels. Separated proteins were then electroblotted onto PVDF membranes (Immobilon P, Millipore, Bedford, MA). Equivalent loadings were verified by Ponceau Red staining after transference. Membranes were blocked in Tris-buffered saline (pH 7.4) containing 0.1% Tween 20 (TBST) and 5% non-fat dried milk for 60 min at room temperature. The blots were then hybridized overnight at 4°C with monoclonal antibodies against the beta chain of HPT (ab13429, 1:500 dilution, Abcam, Cambridge, UK), diluted in TBST with 2% nonfat milk. After thorough washing with TBST, immunoreactive bands were detected by chemiluminescence using the corresponding horseradish peroxidase-conjugated secondary antibodies and ECL detection reagents (GE Healthcare), and then digitized using an LAS 3000 image analyzer. Quantitative changes in band intensities were evaluated with ImageQuant 5.2 software (GE Healthcare). The densitometry values of the Western blot bands containing the HPT were normalized against those of Ponceau staining obtained from the same membranes. Then, the relative abundance of HPT was calculated by obtaining the ratio of the normalized densitometric values between normal and OA samples. Statistical *p* values of the densitometry data were obtained by application of Mann–Whitney *U* test using SPSS version 15.0 program.

## Competing interests

The authors declare that they have no competing interests.

## Authors’ contributions

CF carried out the main experimental work, and prepared the figures and tables. VC participated in the DIGE experiments and gel bioinformatic analyses. PFP collected and classified the serum samples. JLCM participated in study design and helped with the data analysis. CRR participated in the study design, the interpretation of the data and drafted the manuscript. FJB conceived and coordinated the project and revised the manuscript. All authors read and approved the final manuscript.

## Supplementary Material

Additional file 1 **Figure S1.** Representative 2D map of the sequentially depleted serum, with the protein identifications that were carried out. Identifications are listed in Additional file 2: Table S1. Click here for file

Additional file 2 **Table S1.** Mass spectrometry data of the protein identifications performed in the present study. Spot numbers, according to Additional file 1: Figure S1. Click here for file

Additional file 3 **Figure S2.** Amino acid sequence of Haptoglobin, with the MS data corresponding to the identification of alpha and beta chains. Click here for file
