# Supplementary figures and images for: Sequential depletion of human serum for the search of osteoarthritis biomarkers
Source: Proteome Sci. 2012 Sep 12;10:55. doi: 10.1186/1477-5956-10-55 (PMC3515479; doi:10.1186/1477-5956-10-55)

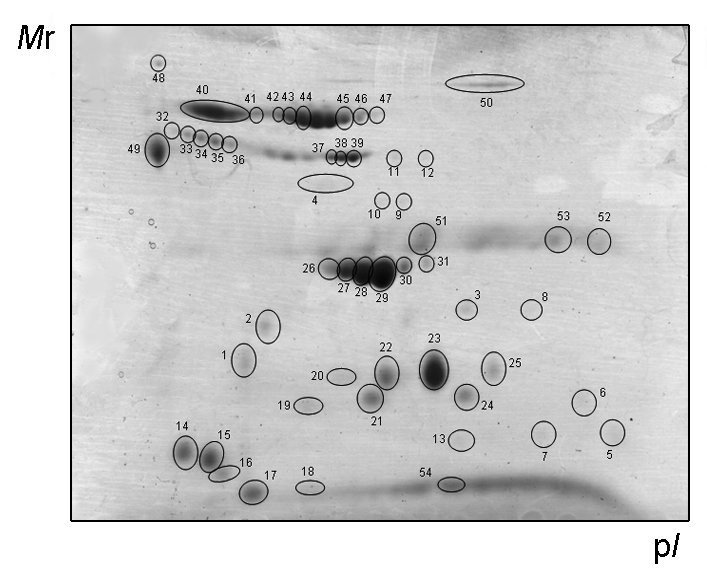

Supplement: Additional file 2 — Table S1. Mass spectrometry data of the protein identifications performed in the present study. Spot numbers, according to Additional file 1: Figure S1. [file 1477-5956-10-55-S1.jpeg]

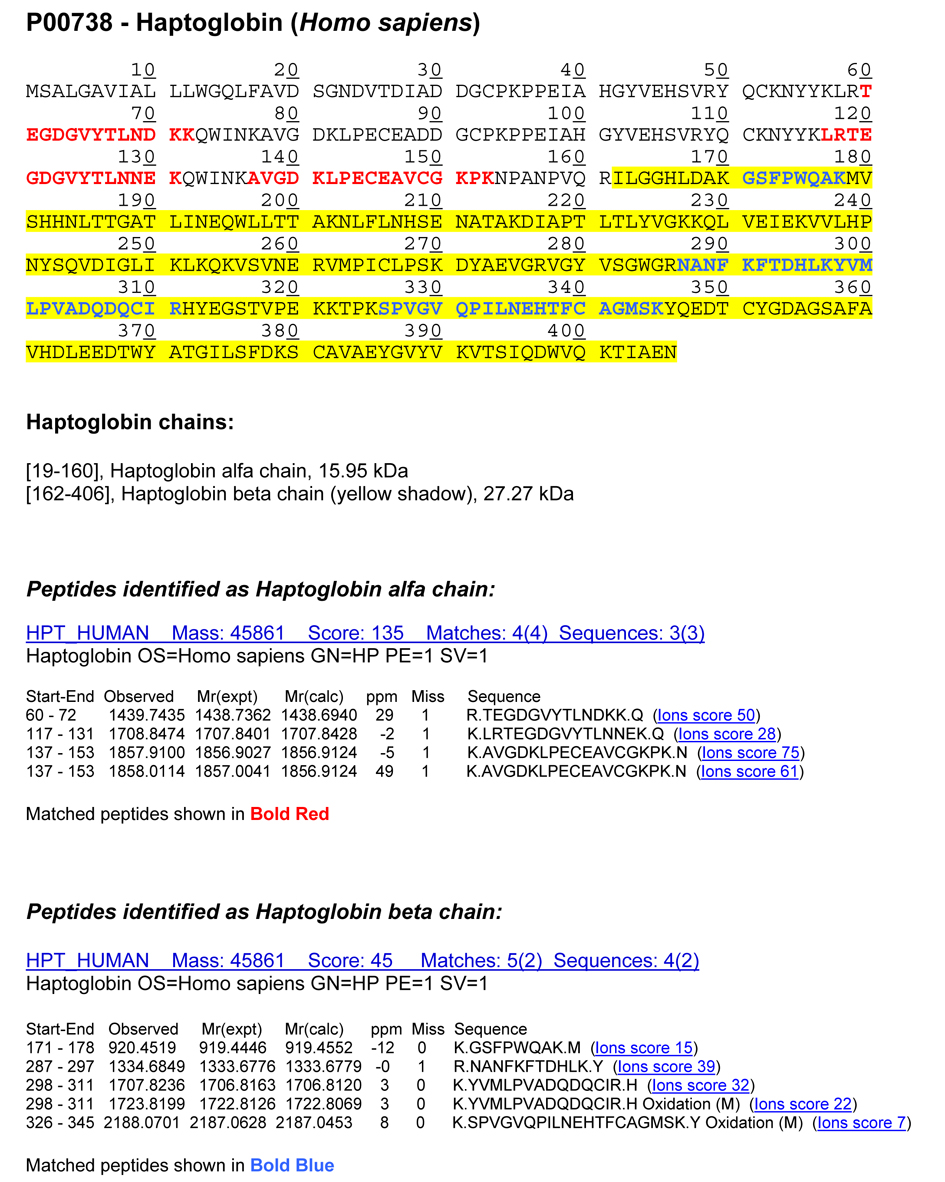

Supplement: Additional file 3 — Figure S2. Amino acid sequence of Haptoglobin, with the MS data corresponding to the identification of alpha and beta chains. [file 1477-5956-10-55-S3.jpeg]
